# Supplementary material for: Cell fixation improves performance of in situ crosslinking mass spectrometry while preserving cellular ultrastructure
Source: Nat Commun. 2024 Oct 2;15:8537. doi: 10.1038/s41467-024-52844-y (PMC11447256; doi:10.1038/s41467-024-52844-y)
Supplement: Supplementary file 1 — Supplementary Information [file 41467_2024_52844_MOESM1_ESM.pdf]

Supplementary Information

Cell fixation improves performance of *in situ* crosslinking mass spectrometry while preserving cellular ultrastructure

Andrew R.M. Michael[1#], Bruno C. Amaral[1#], Kallie L. Ball[1], Kristen H. Eiriksson[1], David C. Schriemer[1,2*]

[1]Department of Biochemistry and Molecular Biology, University of Calgary, Alberta, Canada, T2N-4N1

[2]Department of Chemistry, University of Calgary, Alberta, Canada, T2N-4N1

[#]These authors contribute equally to this work.

*Correspondence to: dschriem@ucalgary.ca

Supplementary Information

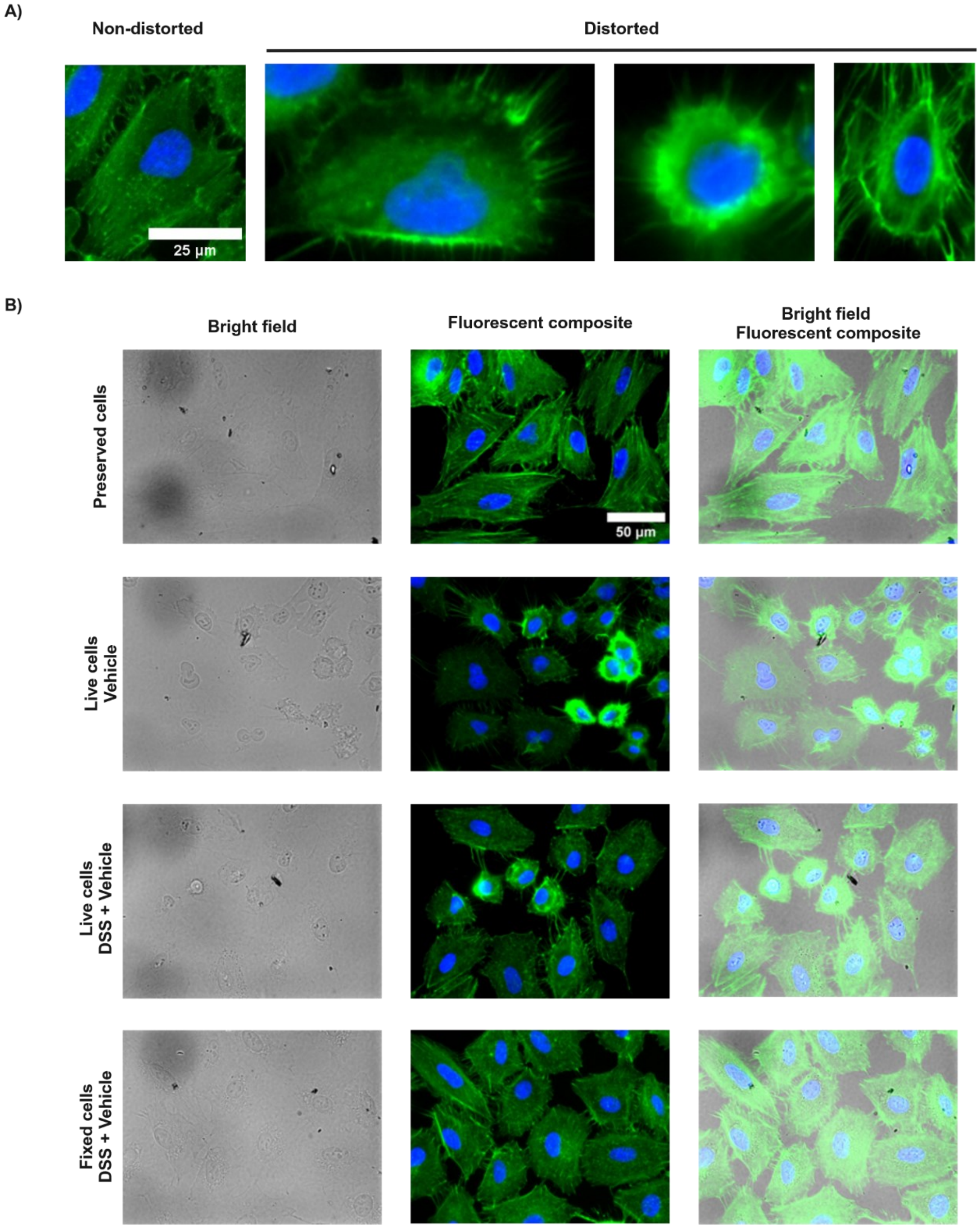

Supplementary Figure 1. Visualization of A549 cells during *in situ* crosslinking. (A) Representative examples of cells for the quantitation of cellular ultrastructure preservation. (B) Bright field and fluorescent images of formaldehyde-preserved cells, live cells treated with DMSO, live cells treated with DSS + DMSO, and formaldehyde-preserved cells treated with DSS + DMSO. Results consistent with 6 similar experiments.

Supplementary Information

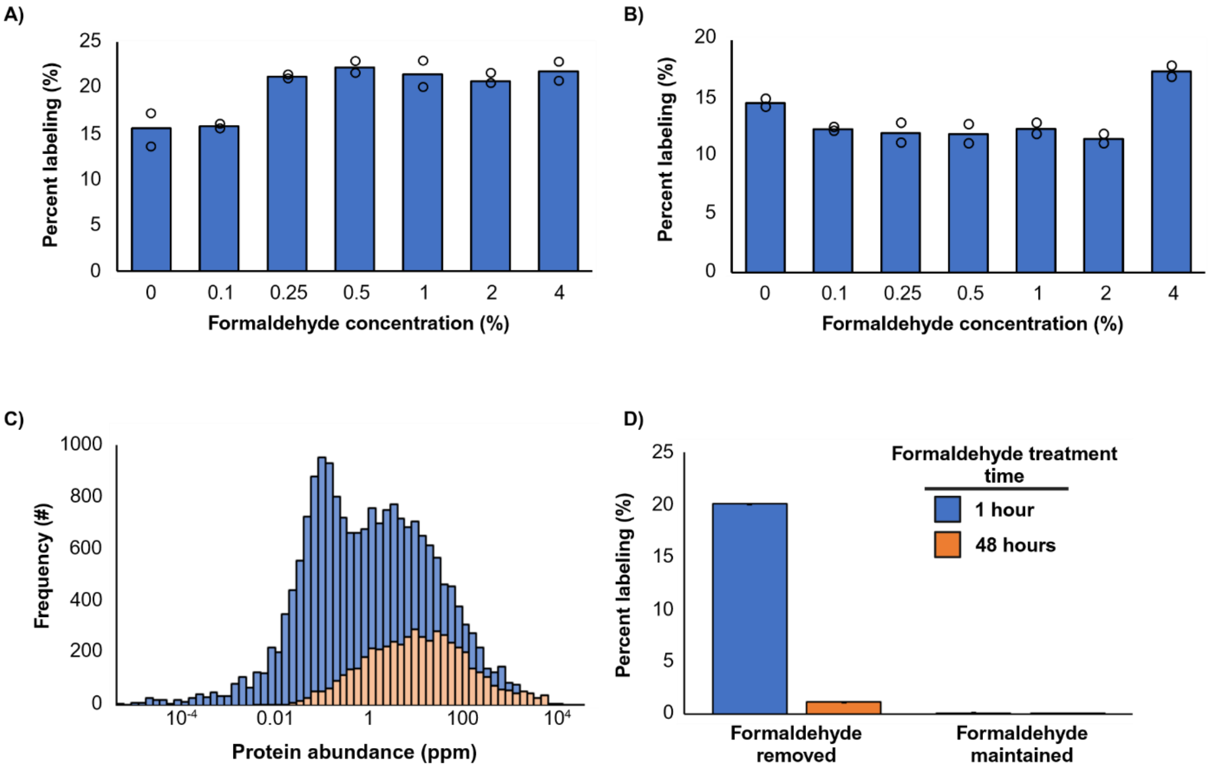

Supplementary Figure 2. NHS-ester labeling in formaldehyde-fixed cells. Labeling analysis of N-(propionyloxy)succinimide reactions with (A) *E. coli* and (B) human cells, over increasing concentrations of formaldehyde ($n = 2$ biological replicates). (C) Protein abundance histogram of human proteome (blue) and protein ID's from MS-acquired non-labeled lysate (orange). Protein abundances retrieved from PaxDb[46]. (D) Labeling of N-(propionyloxy)succinimide in formaldehyde-fixed A549 cells with excess formaldehyde washed away or maintained, after1 hour (blue) or 48 hours (orange) fixation times ($n = 2$ biological replicates).

Supplementary Information

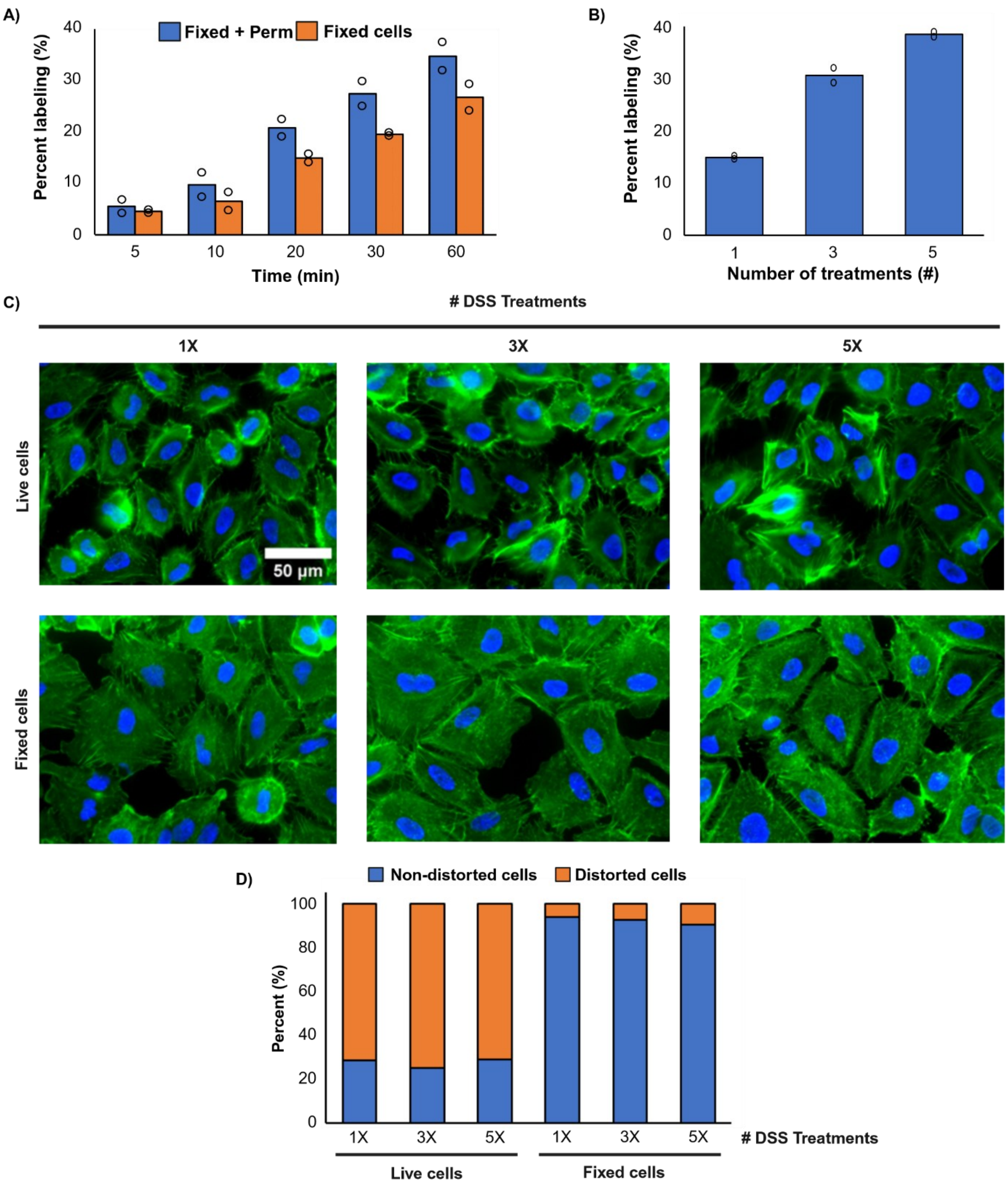

Supplementary Figure 3. Method for increasing labeling yield. (A) Time-course of biotin-X-NHS labeling in A549 cells that have been fixed (orange) or fixed and permeabilized (blue), (n = 2). (B) Percent labeling of increasing number of 1 mM treatments of biotin-X-NHS in fixed A549 cells (n = 2 biological replicates). (C) Fluorescent micrographs of live and fixed A549 cells undergoing increasing numbers of DSS treatments, visualizing actin (green) and DNA (blue). (D) Cells counted for presence (orange) or absence (blue) of cellular disruptions

during increasing numbers of DSS treatments ($n \geq 100$ cells per treatment). Results consistent with a second similar experiment.

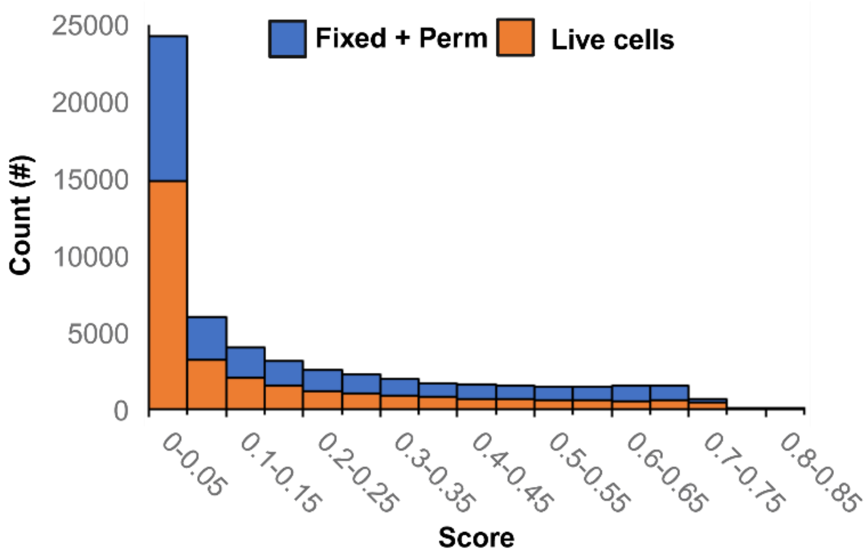

Supplementary Figure 4. CSM score distributions. CSM score distributions of fixed + permeabilized (blue) and Live (orange) DSS treated A549 cells at 5% FDR. Scores obtained from pLink2 data output.

Supplementary Information

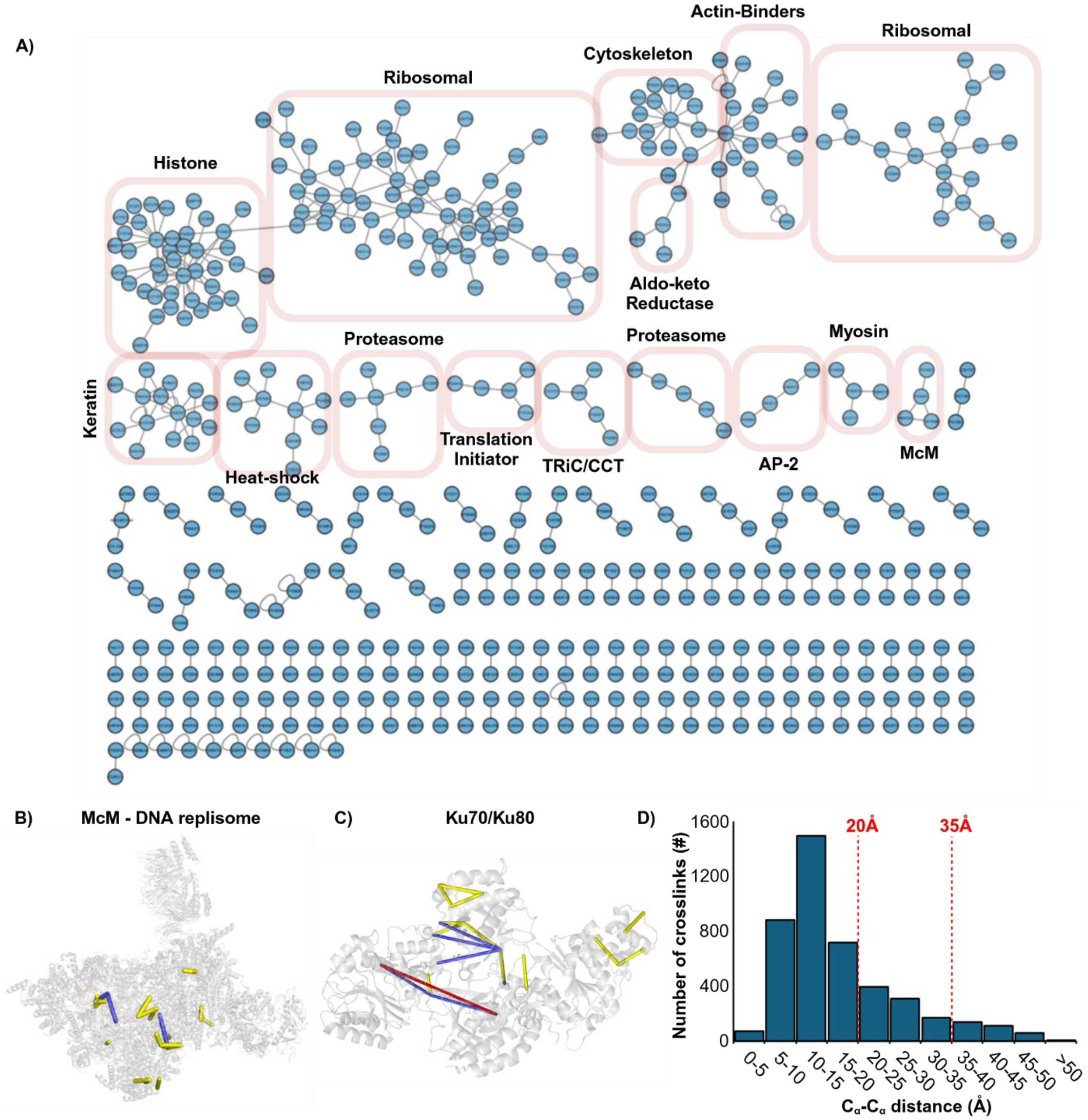

Supplementary Figure 5. ***In situ*** **crosslinking of fixed and permeabilized A549 cells with 1X PhoX.** (A) PPI network plot of all detected interactions from 1X *in situ* PhoX crosslinking. (B) 1X PhoX crosslinks mapped to the McM – DNA replisome (mapped to PDB 7PLO) and (C) Ku70/Ku80 (mapped to PDB 1JEQ). Crosslinks below 20Å, between 20Å and 35Å, and greater than 35Å are coloured yellow, blue, and red, respectively. (D) Histogram of C_α-C_α distances of 1X PhoX crosslinks mapped to all known structures. We note that 73% of the crosslinks are within 20Å and 93% of the crosslinks are within 35Å.

Supplementary Information

Model 1

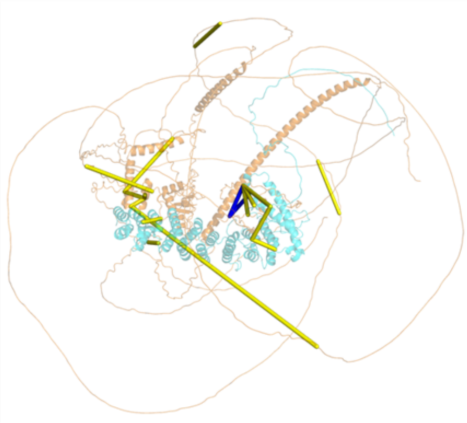

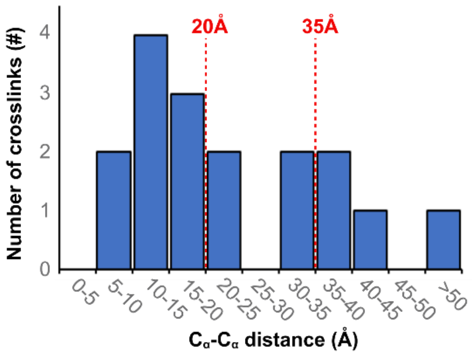

Model 2

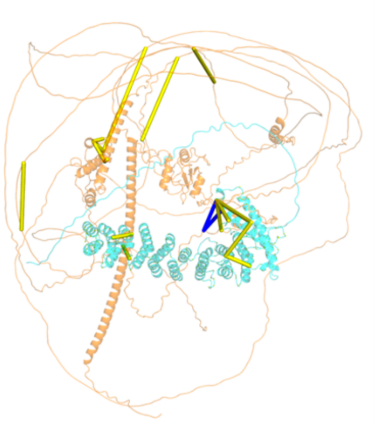

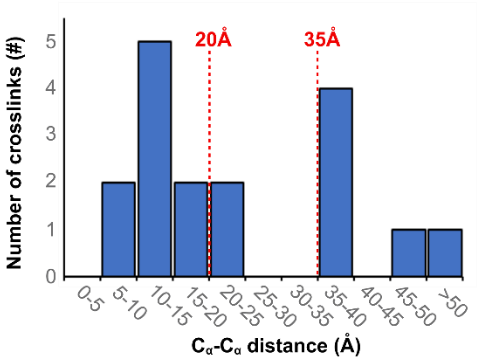

Model 3

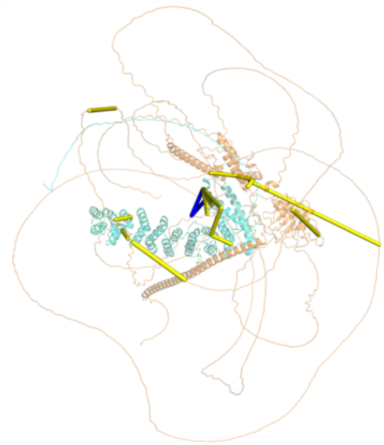

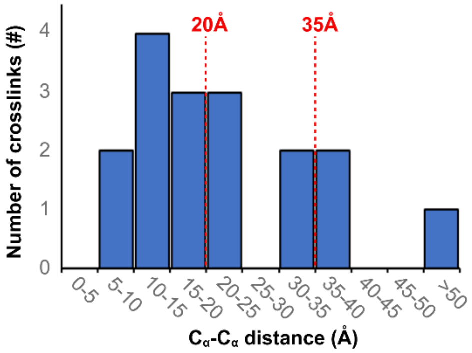

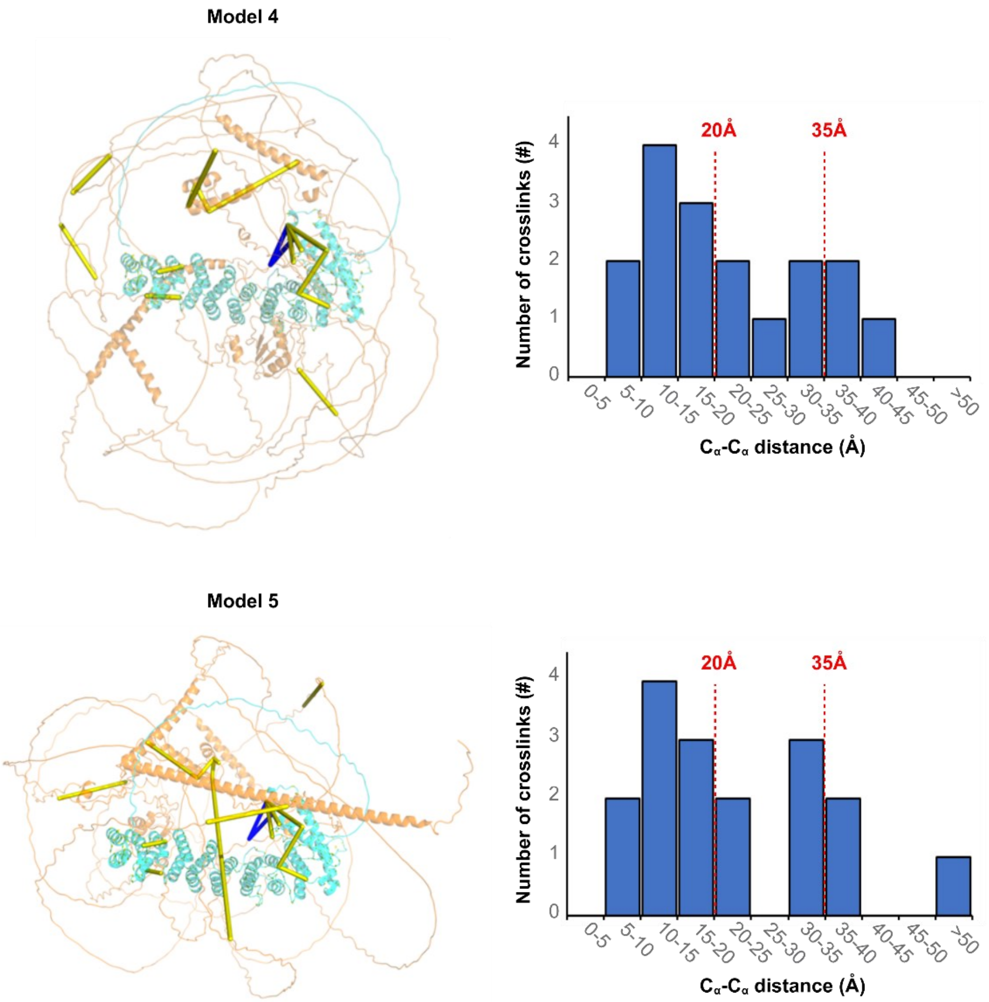

Supplementary Figure 6. Predicted AlphaFold multimer structure of API5-ACIN1 interaction. Crosslinks mapped to 5 predicted models (left) and the corresponding C_α-C_α distances histograms (right) of mapped PhoX crosslinks. API5 and ACIN1 coloured cyan and orange, respectively. Intra-protein and inter-protein crosslinks coloured yellow and blue, respectively.
